# Clearing the Haze: How Does Nicotine Affect Hematopoiesis before and after Birth?

**DOI:** 10.3390/cancers14010184

**Published:** 2021-12-30

**Authors:** Taylor Cool, Alessandra Rodriguez y Baena, E. Camilla Forsberg

**Affiliations:** 1 Program in Molecular, Cell, and Developmental Biology, Institute for the Biology of Stem Cells, University of California Santa Cruz, Santa Cruz, CA 95064, USA; tamccann@ucsc.edu (T.C.); arodr111@ucsc.edu (A.R.y.B.); 2 Institute for the Biology of Stem Cells, Department of Biomolecular Engineering, University of California Santa Cruz, Santa Cruz, CA 95064, USA

**Keywords:** hematopoiesis, hematopoietic stem cells, nicotine, immunity, immune dysfunction, white blood cells

## Abstract

**Simple Summary:**

E-cigarettes have gained popularity as alternatives to traditional tobacco products over the past several decades. Despite being marketed as safer, they still contain several highly toxic compounds, which pose as dangers to human health. Nicotine is one of those toxic compounds and is known to have many deleterious effects on human health and disease susceptibility. Hematopoietic stem cells (HSCs) are the stem cells that give rise to the entire immune system and therefore serve as a compelling point of interrogation for the source of altered health and disease susceptibility in exposed individuals. Here we discuss how nicotine influences HSCs and the immune cells they make, as well as highlight potential mechanisms of altered immunity for life.

**Abstract:**

Hematopoiesis is a tightly regulated process orchestrated by cell-intrinsic and cell-extrinsic cues. Over the past several decades, much effort has been focused on understanding how these cues regulate hematopoietic stem cell (HSC) function. Many endogenous key regulators of hematopoiesis have been identified and extensively characterized. Less is known about the mechanisms of long-term effects of environmental toxic compounds on hematopoietic stem and progenitor cells (HSPCs) and their mature immune cell progeny. Research over the past several decades has demonstrated that tobacco products are extremely toxic and pose huge risks to human health by causing diseases like cancer, respiratory illnesses, strokes, and more. Recently, electronic cigarettes have been promoted as a safer alternative to traditional tobacco products and have become increasingly popular among younger generations. Nicotine, the highly toxic compound found in many traditional tobacco products, is also found in most electronic cigarettes, calling into question their purported “safety”. Although it is known that nicotine is toxic, the pathophysiology of disease in exposed people remains under investigation. One plausible contributor to altered disease susceptibility is altered hematopoiesis and associated immune dysfunction. In this review, we focus on research that has addressed how HSCs and mature blood cells respond to nicotine, as well as identify remaining questions.

## 1. Introduction

An environmental exposure that has become increasingly important in the research field is exposure to tobacco products. According to the National Institute of Health (NIH), one-fourth of the U.S. population uses tobacco products [1]. An abundance of evidence has demonstrated that smoking is highly toxic to human health. Societal use of e-cigarettes has emerged recently and been marketed as a “safer alternative” to traditional tobacco products. Currently, 10 million adults and over 5 million middle and high school students use e-cigarettes in the US [2]. Over the last several decades, researchers have demonstrated that tobacco exposure is not only dangerous to the person using the products, but also to people who are exposed second- or even third-hand [3,4]. Tobacco products are highly complex and made up of several extremely toxic compounds, including hydrogen cyanide, formaldehyde, benzene, nicotine, and more [5]. One of the toxic compounds found in almost all present-day tobacco products is nicotine, a stimulant known for its addictive properties. The oral lethal dose (LD50) of nicotine in humans is ~0.8–1 mg/kg [6,7,8,9]. Nicotine is still highly prevalent in these so-called safer alternatives, presumably to promote addiction to the products. Nicotine has been associated with many deleterious health consequences, including cancer, pulmonary disease, and increased risk of infections [10]. How nicotine influences immunity in first, second, or third-hand exposed people remains an open area of investigation. Interestingly, nicotine has been shown to increase inflammation, as well as white blood cell (WBC) counts [11,12,13,14,15,16]. WBCs are mature, terminally differentiated cells that come from hematopoietic stem cells (HSCs), in a process known as hematopoiesis. WBCs are immune cells with specialized functions, fighting infections and recognizing tumor and diseased cells. Thus, nicotine-induced systemic inflammation and increased WBC count increases the risk for and exacerbates a wide number of clinical conditions, including cancer, cardiovascular disorders, atherosclerosis, autoimmune syndromes, allergy, asthma, and pulmonary disease. The effects that nicotine has on the number and activity of each of these WBC types remains under investigation. This review focuses on the question: what lasting impacts does nicotine have on hematopoiesis and life-long immunity?

## 2. The Hematopoietic Hierarchy

**Figure 1:**
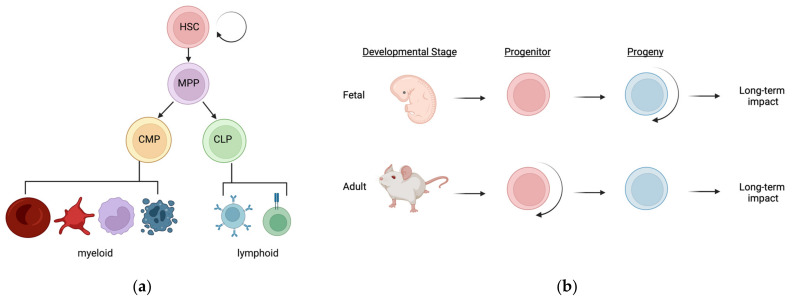
The hematopoietic hierarchy and sources of potential long-term impacts following nicotine exposure: (**a**) Hematopoiesis is the process of generating all mature blood and immune cells from hematopoietic stem cells (HSCs). This process occurs in a well-defined hierarchy during adult steady-state hematopoiesis, as depicted in this simplified tree structure. HSCs differentiate into multipotent progenitors, and then either common myeloid progenitors (CMPs) or common lymphoid progenitors (CLPs), before terminally differentiating into mature blood and immune cells of either myeloid or lymphoid classification. HSCs are unique in their ability to both self-renew as well as differentiate into all of these progenitors and mature cells; (**b**) During fetal hematopoiesis, distinct waves of hematopoietic stem and progenitors (HSPCs) exist throughout development and adulthood. Many of the progenitors that exist during early fetal development are non-self-renewing but can give rise to self-renewing progeny such as “non-traditional” tissue-resident immune cells. Subsequently and during adult steady-state, hematopoiesis is sustained by self-renewing progenitors (HSCs) that give rise to non-self-renewing, short-lived progeny such as “traditional” circulating RBCs and WBCs. Nicotine exposure may influence life-long immunity by two potential mechanisms: (1) nicotine causes changes or persistence in HSPCs which results in altered hematopoietic output for life, or (2) nicotine causes a change in the long-lived immune cells during their establishment which alters immunity later in life. These mechanisms are not mutually exclusive and a combination of both could lead to altered hematopoiesis and altered immunity for life. Adapted from Cool and Forsberg, 2019 [20].

The hematopoietic compartment consists of hematopoietic stem and progenitor cells (HSPCs) and their mature, terminally differentiated progeny (Figure 1a) [17,18]. Hematopoietic stem cells (HSCs) are fascinating because they possess the ability to self-renew as well as differentiate into all mature blood and immune cells (Figure 1a). The hematopoietic process occurs in spatially and temporally distinct waves throughout development, and ultimately gives rise to the complex immune system that patrol all tissues. During fetal development, these distinct waves of HSPCs differentially contribute to mature blood and immune cells across tissues [19,20,21,22]. This complex orchestra of immune layering consists of (1) early developmental, non-self-renewing progenitors that give rise to self-renewing progeny that persist throughout life with little to no contribution from adult HSCs, and (2) subsequently developed self-renewing progenitors (HSCs) that give rise to non-self-renewing progeny that are stably replenished throughout life (Figure 1b). At the interphase of these two well-established paradigms of hematopoietic development exist at least one population of developmentally restricted HSCs (drHSCs) that do not normally self-renew, but can be induced to do so upon transplantation into irradiated hosts [23]. Based on their ability to give rise to robust numbers of traditional as well as atypical lymphoid cells, the physiological role of the drHSCs may be to boost production of lymphoid-mediated immunity that is needed after birth. It is also possible that drHSCs—or other normally transient progenitor cells—are induced to persist for longer time periods upon inflammatory stimulus like nicotine exposure, similar to their induced persistence in an irradiated environment [23,24]. This critical window of perinatal hematopoietic development, with distinct non-persisting stem and progenitor populations contributing alongside “true”, life-long HSCs to a rapidly developing and dynamic immune system, poses as a vulnerable point of interrogation for long-term effects of altered hematopoiesis and immunity in nicotine-exposed individuals. Persisting alterations in cell function can occur in the absence of genetic mutations [25]; the likelihood of lasting physiological responses, however, is greater if cellular changes occur in cells with long half-lives or with self-renewal capacity than in cells with high turnover rates. Potentially, nicotine alters HSCs (or other fetal progenitors) at the epigenetic level, leading to altered HSC function and/or lineage output for life. Alternatively, nicotine alters the numbers and/or epigenetics of mature blood cells, leading to permanently altered immune function (Figure 1b). Whether and how nicotine exposure alters HSCs or their progeny, or both, to cause long-term changes in disease susceptibility remains to be determined.

## 3. Regulation of Hematopoietic Homeostasis

The development of this complex immune system relies on intrinsic and extrinsic cues [26]. Several key regulators of hematopoiesis have been identified and characterized in detail. Transcription factors such as Runx1, SCL, Gata-2, and C-myb play key roles in the intrinsic regulation of HSC potential during development [20,27,28]. Additionally, inflammatory regulators like interferons, interleukin (IL)-1, IL-6, and TNFα provide HSC-extrinsic cues to regulate HSC fate choice and function [29,30,31,32]. Although we have relatively clear and convincing research describing how specific intrinsic and extrinsic regulators influence cell fate choice, many environmental factors that can also significantly impact HSC function and potential have not been thoroughly investigated. Importantly, the timing and duration of these exposures can impact hematopoietic development and function either transiently or for life. While acute exposures can have a transient effect on hematopoiesis, in this review, we focus on the potential mechanisms of altered hematopoiesis and HSC function after chronic challenge. As alluded to above, nicotine may alter long-term immunity via two potential mechanisms: (1) persistent changes in HSCs that then have lasting impacts on hematopoiesis (and immunity) for life, and/or (2) persistent changes in mature immune cells (Figure 1b). In this review, we focus on several studies that have investigated the effects of nicotine on HSCs and WBCs, as well as how nicotine alters inflammatory mediators. We highlight potential mechanisms of altered hematopoiesis and immunity and identify experiments that could help untangle whether these effects are due to changes in HSCs or changes in their mature progeny.

## 4. Does Nicotine Alter Hematopoiesis by Direct Action on HSCs?

**Figure 2:**
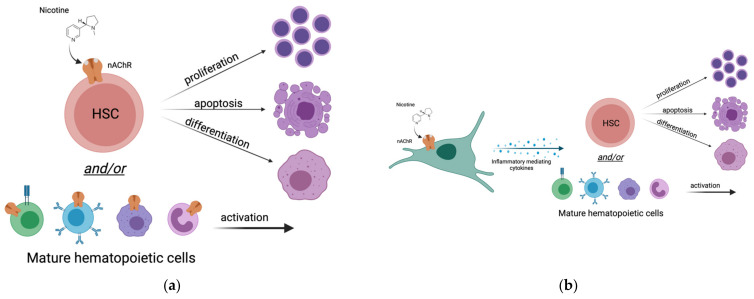
Potential mechanisms of altered hematopoiesis and immunity for life following nicotine exposure: (**a**) Nicotine directly influences the hematopoietic compartment. Nicotine binds to nicotinic-acetyl choline receptors (nAChRs) expressed by hematopoietic stem cells or other mature hematopoietic cell types. These cells then undergo molecular changes which lead to proliferation, apoptosis, differentiation, and/or activation; (**b**) Nicotine indirectly influences the hematopoietic compartment. Nicotine binds to nicotinic-acetyl choline receptors (nAChRs) expressed by non-hematopoietic cells. These cells undergo molecular changes that then lead to release of inflammatory-mediating cytokines. Cytokines released by the non-hematopoietic cells bind to receptors on hematopoietic cell types. Hematopoietic cells then undergo molecular changes in response to the cytokines (directly) and nicotine (indirectly) which leads to proliferation, apoptosis, differentiation, and/or activation.

Tobacco product use is associated with increased WBC counts in peripheral blood which is a sign of increased systemic inflammation [12,13,14,15,16,33,34,35]. Specifically, it is known that nicotine in tobacco products can alter WBC counts in the long-term [11]. However, the mechanism underlying these changes remains unclear. Additionally, whether tobacco product use alters counts of non-traditional immune cells in different tissues has not been investigated. Potentially, there are two plausible mechanisms of altered WBC counts: (1) nicotine directly affects hematopoietic cells by direct binding to nicotinic receptors expressed by HSCs and/or their progeny (Figure 2a), or (2) nicotine indirectly affects the hematopoietic compartment by triggering release of inflammatory-mediating cytokines from non-hematopoietic cells that then act on HSCs and/or their progeny (Figure 2b). It is important to note that these potential mechanisms are not mutually exclusive, and that the effects of nicotine could be a combination of both mechanisms (Figure 2a,b). In this section, we discuss the evidence for nicotine acting as a nicotinic cholinergic receptor agonist and directly affecting hematopoiesis via binding to nicotinic acetylcholine receptor (nAChRs) on hematopoietic cell types.

### 4.1. Do HSCs Express Nicotinic Acetylcholine Receptors (nAChRs)?

One potential mechanism of altered WBCs after nicotine exposure is that nicotine directly affects HSCs via nAChRs expressed on their cell membrane. nAChRs are a family of ligand-gated ion channels. There are 16 homologous subunits identified in mammals, and these subunits combine to form many different nAChR subtypes. Interestingly, these subtypes have various expression patterns across tissues, diverse functional properties and pharmacological characteristics [36,37]. A few groups have provided evidence that HSCs, and some of their mature immune cell progeny, express several subunits of the nAChRs at steady state, and that the expression of other subunits can be induced after exposure to nicotine [11,38]. Chang et al. reported an increase in both HSC and WBC numbers in nicotine-exposed mice, and expression of the nAChR alpha 7 subunit (nAChRα7) in total bone marrow, as well as on isolated HSCs [11]. They used a combination of flow cytometry and immunofluorescence imaging to assess nAChRα7 expression on whole bone marrow (WBM) cells which contain HSPCs and mature cells, as well as on purified HSCs. In this experiment they used the nAChRα7 ligand Alpha Bungarotoxin (abgt) conjugated to a FITC fluorophore to determine whether WBM cells or HSCs expressed the receptor on the cell membrane. Abgt is known to bind with high affinity to the nAChRα7 subunit. Using this method, they determined that some cells within the WBM fraction and purified HSCs both displayed the receptor for abgt ligand. Although they demonstrated that WBM and HSCs had positive staining for the abgt ligand (presumably binding to the nAChRα7 subunit), it should be noted that this assay did not directly determine the levels of nAChRα7 in these cells nor did they include a strong positive control (such as brain homogenate [38]) or negative control (tissue known to not express nAChRα7) to rigorously decipher the relative expression of nAChRα7. An interesting experiment that would have strengthened their findings would be to perform the same experiment with HSCs from wild type (WT) mice and mice lacking nAChRα7. If they had observed that WT HSCs had positive staining for the FITC-abgt, but the mutant HSCs did not, this would more unequivocally have supported their conclusion that HSCs express nAChRα7.

### 4.2. Do Other Hematopoietic Cells Express nAChRs?

In a separate study, St-Pierre et al. took a different approach to address this same question: Do hematopoietic cells express nAChRs [38]? They used freshly isolated murine WBM cells and brain tissue (as a positive control) to perform RT-PCR for the several different subunits of nAChRs. Using this method, they determined that nAChRα9 and nAChRβ2 mRNAs were expressed by nearly all bone marrow cells, while nAChRα7 was expressed only in CD34+ progenitors, monocytes, and B cells. They concluded that long-term HSCs do not actually express the α7 or α9 nAChR subunits, but progenitors and some mature blood cells do. However, one important thing to note about these findings is that they were unable to detect these subunits using qRT-PCR as the detection levels were below threshold at 35 cycles. For this reason, they performed nested RT-PCR instead and observed that nAChR mRNA expression was highly variable across hematopoietic populations. Since mRNA expression does not always result in protein expression, they also investigated whether nicotine could modulate bone marrow-derived myeloid cell numbers via nAChRα7 and nAChRα9 by performing in vitro and in vivo experiments using WT, nAChRα7 knockout (KO), and nAChRα9 KO mice. Interestingly, their in vitro and in vivo experiments provided contradictory results. In vitro, nicotine reduced total numbers of bone marrow-derived monocytes (BMDMs) in WT mice, but not in the two mutant mice. However, in their in vivo model, nicotine had a protective effect on BMDMs. It is important to note that these in vitro and in vivo experiments lacked a nicotine-only control group. A more convincing and straightforward experiment would have been to do a systematic side-by-side comparison of the effects on nicotine on BMDMs in vitro and in vivo in all 3 models (WT, nAChRα7 KO, and nAChRα9 KO) with nicotine only versus control.

Overall, the evidence for robust and functional cell surface expression of nAChR subunits on HSCs and other hematopoietic subsets is not unequivocally convincing. A more direct and definitive approach to determine this would be to purify various hematopoietic cell populations from the murine bone marrow, and test expression by flow cytometry or immunohistochemistry using antibodies specific to each nAChR subunit, with the corresponding cells from gene deletion models serving as controls. If HSCs and mature immune cells do in fact express nAChR subunits on the membrane, it could be assumed that they would *directly* respond to nicotine, at least in part, via binding of the nAChRs expressed. Additionally, in vitro exposure of HSCs and/or their mature immune cell progeny to nicotine-containing media may provide more concrete evidence as to whether nicotine can directly affect these cells, and specifically which ones. At present, a more thorough investigation of the direct effects of nicotine on hematopoietic cell types is needed.

## 5. Does Nicotine Affect Hematopoiesis via an Altered Inflammatory State?

As an alternative to nicotine affecting hematopoiesis through binding nAChRs on hematopoietic cells and/or their progeny, nicotine may potentially affect hematopoiesis indirectly via inflammatory cues (Figure 2). Nicotine is known to induce the release of several inflammatory-mediating cytokines including TNFα, IL-6, IL-1, and others which are also known to play important roles in hematopoietic cell development and homeostasis [32,39,40,41,42,43]. While hematopoietic cells are known to be the source for some of these inflammatory mediators, other cell types may also contribute to altered inflammation, including epithelial cells [44]. In this scenario, one might hypothesize that nicotine acts on non-hematopoietic cells that do express nAChRs, and once the nAChR signaling cascade is initiated in these cell types, the cells undergo molecular changes to respond to the stimulus and can send informative cues (cytokines) to neighboring cell types or into circulation to reach distant tissues and elicit cellular responses [45]. There are several cell types that are known to respond directly to nicotine, including muscle cells and neurons. The vagus nerve is a complex network of neurons that connects the brain with the rest of the organs in the body. It has been recently demonstrated that the vagus nerve, in addition to controlling heart rate, stress, and hormone secretion [46], also acts as an immunomodulator [47]. Coincidentally, acetylcholine is the main neurotransmitter of the vagus nerve, and controls immune cell function via the nAChRα7 subunit. Nicotine, which has a similar structure to acetylcholine, potentially binds to nAChRs to activate the release of inflammatory-mediating cytokines as a form of communication with surrounding organs (Figure 2). Hematopoietic cells (both HSCs and mature immune cells) are known to express receptors for many cytokines [29] and, although they may not be able to directly respond to nicotine, they can therefore undoubtedly respond to many inflammatory cues.

Nicotine leading to an altered inflammatory state has been supported by several studies [33,38,42,45,48,49]. In rodents, it has been demonstrated that nicotine exposure increases the release of pro-inflammatory cytokines. Since ~10% of pregnant women continue smoking during gestation [50], many studies have focused on understanding the effects of in utero nicotine exposure. Similar to data from adults, nicotine is also able to induce inflammation in a developing rodent fetus. Mohsenzadeh et al. exposed pregnant rats to nicotine and measured the serum of their pups after birth to determine concentrations of several inflammatory-mediating cytokines [40]. They found that hs-CRP, IL-6, and TNFα were all elevated in the nicotine-exposed pups compared to their control counterparts. They concluded that in utero nicotine exposure induces a dose-dependent increase in inflammatory-mediating cytokines. Similarly, Orellana et al. observed significantly elevated serum levels of IL-1β and TNFα in the offspring of nicotine-exposed mice [51]. Additionally, day 8.5 mouse embryos exposed to nicotine developed abnormalities and showed increased inflammation by elevated expression of TNFα, IL-1β, and caspase 3 [52]. Another study aimed to determine the effects of in utero nicotine exposure found an increased risk for intrauterine infection and altered inflammatory profile of fetal tissues in rodents [41]. In this study, they measured cytokines from placental tissue and amniotic fluid from nicotine-exposed pregnant dams. Interestingly, they observed that nicotine exposure did not significantly increase TNFα in either tissue. IFNγ was significantly elevated in placental tissue, but significantly decreased in the amniotic fluid of nicotine-exposed fetuses. IL-6, which was unchanged in the nicotine-exposed placental tissues, was significantly increased in amniotic fluid. They concluded that these altered inflammatory mediators were the cause of increased infection susceptibility of the fetus of nicotine-exposed dams. It should be noted that the different outcomes of these studies could be attributed to several factors, including the mode and amount of exposure to nicotine, analysis of different tissues (fetal blood versus placenta/amniotic fluid), and time of tissue collection (newborn rats versus gestational day 18). These studies also only probed a few cytokines and did not investigate the underlying mechanism of altered inflammation.

Overall, these studies indicate that the exposure of pregnant females to nicotine can lead to an altered inflammatory environment during gestation, which may have important consequences on fetal hematopoiesis [29,53,54]. Moreover, it is yet unknown whether this is caused directly by nicotine in the fetal environment or by passage of maternal nicotine-induced inflammatory cytokines to the fetus during development. To understand the mechanism of altered inflammation in nicotine-exposed pups, it would be necessary to identify the cells to which nicotine is binding and initiating signaling, and then determining if those cells are the sole source of cytokines or if they work in concert with other cells to elicit an immune response. Although we know that nicotine can cross the placenta and accumulates in the fetal blood and in the breast milk [55,56,57,58,59], it remains unclear whether these significant fetal and neonatal exposures lead to direct changes in the fetal hematopoietic compartment. It is also unknown whether nicotine leads to transient or persisting alterations in developmental hematopoiesis.

## 6. Conclusions

Persisting alterations to hematopoiesis may serve as a mechanism of disease susceptibility later in life. Although it is widely agreed that nicotine is extremely toxic, the mechanism of altered immunity of nicotine-exposed individuals remains a topic of intense investigation. The emergence of e-cigarettes as alternatives to traditional tobacco products has prompted a new wave of health concerns and a need for scientific research into the effects of their toxic components on human health. Nicotine is highly addictive and found in almost all present-day tobacco products, new and old, and therefore serves as a logical starting point of investigation of the toxic effects of tobacco product use on human health. Decades of research have demonstrated that children of smoking mothers have diminished health [59,60,61], yet the mechanism of altered disease susceptibility remains unclear. As the hematopoietic system is the focal point of immunity and health, the effects of nicotine on hematopoietic cell types warrant further investigation. Nicotine potentially *directly* affects hematopoietic cells, including HSCs, via binding their nAChRs. Direct action on HSCs is consistent with the increase in HSC numbers and detection of nAChRα7 expression on the surface of HSCs [11]. Alternatively, nicotine may *indirectly* affect hematopoietic cell types, by binding nAChRs on other cell types (likely epithelial, neuronal, muscular cells) that then secrete cytokines to induce inflammation. A third possibility is that nicotine affects hematopoiesis both directly and indirectly, leading to feedback loops that perpetuate inflammation. In order to advance our understanding of the effects of nicotine on hematopoietic cell types and immunity, thorough investigation into these possible mechanisms is needed. Experiments with genetic deletion or gain-of-function models, possibly facilitated by the many rapidly emerging CRISPR technologies [62,63], should enable unequivocal new results. Exciting approaches to move the field forward are increasingly feasible. For example, the effect of nicotine on mature immune cell subsets could be assessed at high resolution by single-cell RNA sequencing, possibly revealing alterations of activation genes in T cells. Analogously, ATAC-seq, of bulk or single cells [64,65], could be implemented to test the hypothesis that fetal and/or adult HSCs have altered functional output in response to nicotine due to lasting epigenetic changes. The discovery of drHSCs [23] and improved characterization of non-traditional immune cells [66,67,68,69] opens new and intriguing avenues of exploration. Together with a systematic investigation of the direct and indirect effects of nicotine on hematopoiesis, these strategies will provide insights needed to understand and mitigate damage to its exposure.

## References

[1] National Institute on Drug Abuse What is the Scope of Tobacco, Nicotine, and E-Cigarette Use in the United States?. www.drugabuse.gov/publications/research-reports/tobacco-nicotine-e-cigarettes/what-scope-tobacco-use-its-cost-to-society.

[2] Ramanathan G., Craver-Hoover B., Arechavala R.J., Herman D.A., Chen J.H., Lai H.Y., Renusch S.R., Kleinman M.T., Fleischman A.G. (2020). E-Cigarette Exposure Decreases Bone Marrow Hematopoietic Progenitor Cells. Cancers.

[3] Flouris A.D., Vardavas C.I., Metsios G.S., Tsatsakis A.M., Koutedakis Y. (2010). Biological Evidence for the Acute Health Effects of Secondhand Smoke Exposure. Am. J. Physiol. Lung Cell. Mol. Physiol..

[4] Ferrante G., Simoni M., Cibella F., Ferrara F., Liotta G., Malizia V., Corsello G., Viegi G., La Grutta S. (2015). Third-Hand Smoke Exposure and Health Hazards in Children. Monaldi Arch. Chest Dis..

[5] Harmful and Potentially Harmful Constituents in Tobacco Products and Tobacco Smoke: Established List. https://www.fda.gov/tobacco-products/rules-regulations-and-guidance/harmful-and-potentially-harmful-constituents-tobacco-products-and-tobacco-smoke-established-list.

[6] Mayer B. (2014). How Much Nicotine Kills a Human? Tracing Back the Generally Accepted Lethal Dose to Dubious Self-Experiments in the Nineteenth Century. Arch. Toxicol..

[7] Thornton S.L., Oller L., Sawyer T. (2014). Fatal Intravenous Injection of Electronic Nicotine Delivery System Refilling Solution. J. Med. Toxicol..

[8] Sommerfeld K., Łukasik-Głębocka M., Kulza M., Drużdż A., Panieński P., Florek E., Zielińska-Psuja B. (2016). Intravenous and Oral Suicidal E-Liquid Poisonings with Confirmed Nicotine and Cotinine Concentrations. Forensic Sci. Int..

[9] Belkoniene M., Socquet J., Njemba-Freiburghaus D., Pellaton C. (2019). Near Fatal Intoxication by Nicotine and Propylene Glycol Injection: A Case Report of an e-Liquid Poisoning. BMC Pharmacol. Toxicol..

[10] Mishra A., Chaturvedi P., Datta S., Sinukumar S., Joshi P., Garg A. (2015). Harmful Effects of Nicotine. Indian J. Med. Paediatr. Oncol..

[11] Chang E., Forsberg E.C., Wu J., Bingyin Wang B., Prohaska S.S., Allsopp R., Weissman I.L., Cooke J.P. (2010). Cholinergic Activation of Hematopoietic Stem Cells: Role in Tobacco-Related Disease?. Vasc. Med..

[12] Zalokar J.B., Richard J.L., Claude J.R. (1981). Leukocyte Count, Smoking, and Myocardial Infarction. N. Engl. J. Med..

[13] Smith M.R., Kinmonth A.-L., Luben R.N., Bingham S., Day N.E., Wareham N.J., Welch A., Khaw K.-T. (2003). Smoking Status and Differential White Cell Count in Men and Women in the EPIC-Norfolk Population. Atherosclerosis.

[14] Friedman G.D., Siegelaub A.B., Seltzer C.C., Feldman R., Collen M.F. (1973). Smoking Habits and the Leukocyte Count. Arch. Environ. Health Int. J..

[15] Roethig H.J., Koval T., Muhammad-Kah R., Jin Y., Mendes P., Unverdorben M. (2010). Short Term Effects of Reduced Exposure to Cigarette Smoke on White Blood Cells, Platelets and Red Blood Cells in Adult Cigarette Smokers. Regul. Toxicol. Pharmacol..

[16] Fernández J.A., Prats J., Artero J.V., Mora A., Fariñas A., Espinal A., Méndez J.A. (2012). Systemic Inflammation in 222.841 Healthy Employed Smokers and Nonsmokers: White Blood Cell Count and Relationship to Spirometry. Tob. Induc. Dis..

[17] Rodriguez y Baena A., Manso B.A., Forsberg E.C. (2021). CFU-S Assay: A Single-Cell Historical Assay Offers Modern Insight to Clonal Hematopoiesis. Exp. Hematol..

[18] Till J.E., Mcculloch E.A. (1961). A Direct Measurement of the Radiation Sensitivity of Normal Mouse Bone Marrow Cells. Radiat. Res..

[19] Dzierzak E., Bigas A. (2018). Blood Development: Hematopoietic Stem Cell Dependence and Independence. Cell Stem Cell.

[20] Cool T., Forsberg E.C. (2019). Chasing Mavericks: The Quest for Defining Developmental Waves of Hematopoiesis. Current Topics in Developmental Biology.

[21] Klaus A., Robin C. (2017). Embryonic Hematopoiesis under Microscopic Observation. Dev. Biol..

[22] Wittamer V., Bertrand J.Y. (2020). Yolk Sac Hematopoiesis: Does It Contribute to the Adult Hematopoietic System?. Cell. Mol. Life Sci..

[23] Beaudin A.E., Boyer S.W., Perez-Cunningham J., Hernandez G.E., Derderian S.C., Jujjavarapu C., Aaserude E., MacKenzie T., Forsberg E.C. (2016). A Transient Developmental Hematopoietic Stem Cell Gives Rise to Innate-like B and T Cells. Cell Stem Cell.

[24] Beaudin A.E., Forsberg E.C. (2016). To B1a or Not to B1a: Do Hematopoietic Stem Cells Contribute to Tissue-Resident Immune Cells?. Blood.

[25] De Laval B., Maurizio J., Kandalla P.K., Brisou G., Simonnet L., Huber C., Gimenez G., Matcovitch-Natan O., Reinhardt S., David E. (2020). C/EBPβ-Dependent Epigenetic Memory Induces Trained Immunity in Hematopoietic Stem Cells. Cell Stem Cell.

[26] Rodrigues C.P., Shvedunova M., Akhtar A. (2021). Epigenetic Regulators as the Gatekeepers of Hematopoiesis. Trends Genet..

[27] Wang Z., Wang P., Li Y., Peng H., Zhu Y., Mohandas N., Liu J. (2021). Interplay between Cofactors and Transcription Factors in Hematopoiesis and Hematological Malignancies. Sig. Transduct. Target. Ther..

[28] Gao P., Chen C., Howell E.D., Li Y., Tober J., Uzun Y., He B., Gao L., Zhu Q., Siekmann A.F. (2020). Transcriptional Regulatory Network Controlling the Ontogeny of Hematopoietic Stem Cells. Genes Dev..

[29] Pietras E.M. (2017). Inflammation: A Key Regulator of Hematopoietic Stem Cell Fate in Health and Disease. Blood.

[30] Schuettpelz L.G., Link D.C. (2013). Regulation of Hematopoietic Stem Cell Activity by Inflammation. Front. Immunol..

[31] Baldridge M.T., King K.Y., Goodell M.A. (2011). Inflammatory Signals Regulate Hematopoietic Stem Cells. Trends Immunol..

[32] Collins A., Mitchell C.A., Passague E. (2021). Inflammatory Signaling Regulates Hematopoietic Stem and Progenitor Cell Development and Homeostasis. J. Exp. Med..

[33] Flouris A.D., Poulianiti K.P., Chorti M.S., Jamurtas A.Z., Kouretas D., Owolabi E.O., Tzatzarakis M.N., Tsatsakis A.M., Koutedakis Y. (2012). Acute Effects of Electronic and Tobacco Cigarette Smoking on Complete Blood Count. Food Chem. Toxicol..

[34] Pedersen K.M., Çolak Y., Ellervik C., Hasselbalch H.C., Bojesen S.E., Nordestgaard B.G. (2019). Smoking and Increased White and Red Blood Cells: A Mendelian Randomization Approach in the Copenhagen General Population Study. Arterioscler. Thromb. Vasc. Biol..

[35] Chmielewski P.P., Strzelec B. (2018). Elevated Leukocyte Count as a Harbinger. Folia Morphol..

[36] Dani J.A. (2015). Neuronal Nicotinic Acetylcholine Receptor Structure and Function and Response to Nicotine. International Review of Neurobiology.

[37] Wu J., Lukas R.J. (2011). Naturally-Expressed Nicotinic Acetylcholine Receptor Subtypes. Biochem. Pharmacol..

[38] St-Pierre S., Jiang W., Roy P., Champigny C., LeBlanc É., Morley B.J., Hao J., Simard A.R. (2016). Nicotinic Acetylcholine Receptors Modulate Bone Marrow-Derived pro-Inflammatory Monocyte Production and Survival. PLoS ONE.

[39] Osgoei L.T., Parivar K., Ebrahimi M., Mortaz E. (2018). Nicotine Modulates the Release of Inflammatory Cytokines and Expression of TLR2, TLR4 of Cord Blood Mononuclear Cells. Iran. J. Allergy Asthma Immunol..

[40] Mohsenzadeh Y., Rahmani A., Cheraghi J., Pyrani M., Asadollahi K. (2014). Prenatal Exposure to Nicotine in Pregnant Rat Increased Inflammatory Marker in Newborn Rat. Mediat. Inflamm..

[41] Von Chamier M., Reyes L., Hayward L.F., Brown M.B. (2017). Impact of Gestational Nicotine Exposure on Intrauterine and Fetal Infection in a Rodent Model. Biol. Reprod..

[42] Chen H., Li G., Chan Y.L., Chapman D.G., Sukjamnong S., Nguyen T., Annissa T., McGrath K.C., Sharma P., Oliver B.G. (2018). Maternal E-Cigarette Exposure in Mice Alters DNA Methylation and Lung Cytokine Expression in Offspring. Am. J. Respir. Cell Mol. Biol..

[43] Hosseinzadeh A., Thompson P.R., Segal B.H., Urban C.F. (2016). Nicotine Induces Neutrophil Extracellular Traps. J. Leukoc. Biol..

[44] Strzelak A., Ratajczak A., Adamiec A., Feleszko W. (2018). Tobacco Smoke Induces and Alters Immune Responses in the Lung Triggering Inflammation, Allergy, Asthma and Other Lung Diseases: A Mechanistic Review. Int. J. Environ. Res. Public Health.

[45] Hajiasgharzadeh K., Sadigh-Eteghad S., Mansoori B., Mokhtarzadeh A., Shanehbandi D., Doustvandi M.A., Asadzadeh Z., Baradaran B. (2019). Alpha7 Nicotinic Acetylcholine Receptors in Lung Inflammation and Carcinogenesis: Friends or Foes?. J. Cell. Physiol..

[46] Breit S., Kupferberg A., Rogler G., Hasler G. (2018). Vagus Nerve as Modulator of the Brain-Gut Axis in Psychiatric and Inflammatory Disorders. Front. Psychiatry.

[47] Johnston G.R., Webster N.R. (2009). Cytokines and the Immunomodulatory Function of the Vagus Nerve. Br. J. Anaesth..

[48] Wong M.K., Barra N.G., Alfaidy N., Hardy D.B., Holloway A.C. (2015). Adverse Effects of Perinatal Nicotine Exposure on Reproductive Outcomes. Reproduction.

[49] Gracia M.C. (2005). Exposure to Nicotine Is Probably a Major Cause of Inflammatory Diseases among Non-Smokers. Med. Hypotheses.

[50] Azagba S., Manzione L., Shan L., King J. (2020). Trends in Smoking during Pregnancy by Socioeconomic Characteristics in the United States, 2010–2017. BMC Pregnancy Childbirth.

[51] Orellana J.A., Busso D., RamÃ rez G., Campos M., Rigotti A., EugenÃ n J., von Bernhardi R. (2014). Prenatal Nicotine Exposure Enhances Cx43 and Panx1 Unopposed Channel Activity in Brain Cells of Adult Offspring Mice Fed a High-Fat/Cholesterol Diet. Front. Cell. Neurosci..

[52] Lin C., Yon J.-M., Hong J.T., Lee J.K., Jeong J., Baek I.-J., Lee B.J., Yun Y.W., Nam S.-Y. (2014). 4-O-Methylhonokiol Inhibits Serious Embryo Anomalies Caused by Nicotine via Modulations of Oxidative Stress, Apoptosis, and Inflammation: 4-O-METHYLHONOKIOL PREVENTS NICOTINE-INDUCED EMBRYOTOXICITY. Birth Defects Res. B.

[53] Apostol A.C., Jensen K.D.C., Beaudin A.E. (2020). Training the Fetal Immune System through Maternal Inflammation—A Layered Hygiene Hypothesis. Front. Immunol..

[54] Hayashi Y., Sezaki M., Takizawa H. (2019). Development of the Hematopoietic System: Role of Inflammatory Factors. WIREs Dev. Biol..

[55] Qu W., Liu H., Yan H., Hou L., Ping J., Zhao W., Wen X. (2018). Prenatal Nicotine Exposure Induces Thymic Hypoplasia in Mice Offspring from Neonatal to Adulthood. Toxicol. Lett..

[56] Kang N. (2017). Effects of in Utero Nicotine Exposure on Immune Cell Disposition after *P. Aeruginosa* Lung Infection. Master’s Thesis.

[57] Luck W., Nau H. (1984). Nicotine and Cotinine Concentrations in Serum and Milk of Nursing Smokers. Br. J. Clin. Pharmacol..

[58] Dahlström A., Lundell B., Curvall M., Thapper L. (1990). Nicotine and Cotinine Concentrations in the Nursing Mother and Her Infant. Acta Paediatr..

[59] Bruin J.E., Gerstein H.C., Holloway A.C. (2010). Long-Term Consequences of Fetal and Neonatal Nicotine Exposure: A Critical Review. Toxicol. Sci..

[60] Hofhuis W., de Jongste J.C., Merkus P.J.F.M. (2003). Adverse Health Effects of Prenatal and Postnatal Tobacco Smoke Exposure on Children. Arch. Dis. Child..

[61] Wen X., Shenassa E.D., Paradis A.D. (2013). Maternal Smoking, Breastfeeding, and Risk of Childhood Overweight: Findings from a National Cohort. Matern. Child Health J..

[62] González-Romero E., Martínez-Valiente C., García-Ruiz C., Vázquez-Manrique R.P., Cervera J., Sanjuan-Pla A. (2019). CRISPR to Fix Bad Blood: A New Tool in Basic and Clinical Hematology. Haematologica.

[63] Nidhi S., Anand U., Oleksak P., Tripathi P., Lal J.A., Thomas G., Kuca K., Tripathi V. (2021). Novel CRISPR–Cas Systems: An Updated Review of the Current Achievements, Applications, and Future Research Perspectives. Int. J. Mol. Sci..

[64] Buenrostro J.D., Wu B., Chang H.Y., Greenleaf W.J. (2015). ATAC-seq: A Method for Assaying Chromatin Accessibility Genome-Wide. Curr. Protoc. Mol. Biol..

[65] Buenrostro J.D., Giresi P.G., Zaba L.C., Chang H.Y., Greenleaf W.J. (2013). Transposition of Native Chromatin for Fast and Sensitive Epigenomic Profiling of Open Chromatin, DNA-Binding Proteins and Nucleosome Position. Nat. Methods.

[66] Merad M., Manz M.G., Karsunky H., Wagers A., Peters W., Charo I., Weissman I.L., Cyster J.G., Engleman E.G. (2002). Langerhans Cells Renew in the Skin throughout Life under Steady-State Conditions. Nat. Immunol..

[67] Leung G.A., Cool T., Valencia C.H., Worthington A., Beaudin A.E., Forsberg E.C. (2019). The Lymphoid-Associated Interleukin 7 Receptor (IL7R) Regulates Tissue-Resident Macrophage Development. Development.

[68] Cool T., Worthington A., Poscablo D., Hussaini A., Forsberg E.C. (2020). Interleukin 7 Receptor Is Required for Myeloid Cell Homeostasis and Reconstitution by Hematopoietic Stem Cells. Exp. Hematol..

[69] Worthington A., Cool T., Poscablo D., Hussaini A., Beaudin A.E., Forsberg E.C. (2022). IL7R⍺, but not Flk2/Flt3, is required for hematopoietic stem cell reconstitution of tissue-resident lymphoid cells. Development.

